# Advances in the Evaluation of Respiratory Pathophysiology during Exercise in Chronic Lung Diseases

**DOI:** 10.3389/fphys.2017.00082

**Published:** 2017-02-22

**Authors:** Denis E. O'Donnell, Amany F. Elbehairy, Danilo C. Berton, Nicolle J. Domnik, J. Alberto Neder

**Affiliations:** ^1^Division of Respiratory Medicine, Department of Medicine, Queen's University and Kingston General HospitalKingston, ON, Canada; ^2^Department of Chest Diseases, Faculty of Medicine, Alexandria UniversityAlexandria, Egypt

**Keywords:** exercise, dyspnea, pulmonary mechanics, chronic obstructive pulmonary disease, interstitial lung disease, pulmonary vascular diseases

## Abstract

Dyspnea and exercise limitation are among the most common symptoms experienced by patients with various chronic lung diseases and are linked to poor quality of life. Our understanding of the source and nature of perceived respiratory discomfort and exercise intolerance in chronic lung diseases has increased substantially in recent years. These new mechanistic insights are the primary focus of the current review. Cardiopulmonary exercise testing (CPET) provides a unique opportunity to objectively evaluate the ability of the respiratory system to respond to imposed incremental physiological stress. In addition to measuring aerobic capacity and quantifying an individual's cardiac and ventilatory reserves, we have expanded the role of CPET to include evaluation of symptom intensity, together with a simple “non-invasive” assessment of relevant ventilatory control parameters and dynamic respiratory mechanics during standardized incremental tests to tolerance. This review explores the application of the new advances in the clinical evaluation of the pathophysiology of exercise intolerance in chronic obstructive pulmonary disease (COPD), chronic asthma, interstitial lung disease (ILD) and pulmonary arterial hypertension (PAH). We hope to demonstrate how this novel approach to CPET interpretation, which includes a quantification of activity-related dyspnea and evaluation of its underlying mechanisms, enhances our ability to meaningfully intervene to improve quality of life in these pathologically-distinct conditions.

## Introduction

Dyspnea and exercise intolerance are commonly the most troublesome symptoms reported by patients with chronic pulmonary diseases and contribute significantly to poor quality of life. Moreover, dyspnea, physical inactivity and reduced peak oxygen consumption ($\dot{\text{V}}$O_2_) are closely inter-related and have been shown to predict earlier mortality in various chronic pulmonary diseases (Oga et al., 2003; Pinto-Plata et al., 2004; Waschki et al., 2011; Ley et al., 2016). Not surprisingly, improving dyspnea and exercise tolerance are major goals in the management of chronic lung diseases.

Exercise capacity cannot reliably be predicted in any individual based solely on careful clinical assessment or resting pulmonary function tests (O'Donnell et al., 2001). Patients with chronic dyspnea routinely avoid activities that provoke this unpleasant symptom and therefore, commonly underestimate (and under-report) true symptom severity and its negative long-term impact on exercise capacity. Cardiopulmonary exercise testing (CPET) alone provides a rigorous evaluation of the interface between respiratory impairment (caused by disease) and reduced exercise capacity in an individual under measured physiological stress. It uniquely permits an objective assessment of the integrated functions of the neurosensory, metabolic, respiratory, cardiovascular, and locomotor muscle systems to graded physical exertion.

A fact that has traditionally been overlooked in studies of exercise physiology is that the proximate limitation of exercise performance in chronic lung disease populations is very often intolerable symptoms such as dyspnea, not critical encroachment on physiological maxima of the cardiovascular and respiratory systems (Killian et al., 1992; O'Donnell and Webb, 1993; Hamilton et al., 1996). Accordingly, there is renewed interest in elucidating the underlying mechanisms of exertional dyspnea. Moreover, there is now broader acknowledgment that measurements of exertional symptoms and non-invasive dynamic respiratory mechanics are integral components of CPET (ERS Task Force et al., 2007; O'Donnell et al., 2009; Guenette et al., 2013; Puente-Maestu et al., 2016).

For the pulmonologist interested in evaluating the severity of activity-related dyspnea and in discovering its cause(s) in individual patients, we suggest a simple, ordered interrogation of perceptual and physiological responses to incremental exercise. These include: (1) *perceptual responses*: dyspnea (Borg) ratings as a function of increasing work rate (WR) [and/or minute ventilation ($\dot{\text{V}}$_E_)]; (2) *ventilatory control*: $\dot{\text{V}}$_E_-WR, $\dot{\text{V}}$_E_-carbon dioxide production ($\dot{\text{V}}$CO_2_) ratio, O_2_ saturation, and partial pressure of end-tidal carbon dioxide (PetCO_2_) as a function of WR; (3) *dynamic respiratory mechanics*: change in inspiratory capacity (IC), inspiratory reserve volume (IRV), tidal volume (V_T_) and breathing frequency (*f*), all as a function of increasing WR (or $\dot{\text{V}}$_E_); quantitative flow-volume loop analysis (mentioned only briefly in the current review) and (4) *metabolic* and c*ardiocirculatory responses*: $\dot{\text{V}}$O_2_-WR, $\dot{\text{V}}$CO_2_-$\dot{\text{V}}$O_2_ (“V-slope” method to estimate the lactate threshold), heart rate (HR) and O_2_ pulse as a function of $\dot{\text{V}}$O_2_ (Weisman and Zeballos, 1994; Johnson et al., 1999; Ofir et al., 2008a; Chin et al., 2013; Guenette et al., 2014; Elbehairy et al., 2015a,b; Faisal et al., 2015, 2016).

We begin by briefly reviewing the natural changes in respiratory physiology that negatively impact exercise capacity with advancing age, since the healthy elderly are the appropriate reference population for most patients with chronic respiratory conditions. We then describe the abnormal responses to exercise in three separate lung disease categories (obstructive, restrictive and pulmonary vascular diseases). In so doing, we uncover common mechanisms of dyspnea and exercise intolerance across these diverse diseases. Finally, we hope to demonstrate that a simple systematic approach that emphasizes both perceptual and physiological responses (ventilatory control and mechanics) allows the clinician to develop a cogent physiological rationale for effective treatment of dyspnea and exercise tolerance in these common chronic respiratory diseases.

## Older healthy individuals

### Respiratory responses to exercise in older healthy individuals

The healthy respiratory system admirably fulfills its primary task of ensuring that alveolar ventilation ($\dot{\text{V}}$_A_) is commensurate with the increasing muscular metabolic demands of incremental exercise, even at high intensities (West, 2004). Moreover, it accomplishes this feat while maintaining arterial blood gas and acid-base homeostasis and ensuring minimal perceived breathing difficulty. Young, untrained adults can accomplish high peak $\dot{\text{V}}$_E_ (e.g., 120 L/min) with little respiratory discomfort while requiring only 5–7% of their total body $\dot{\text{V}}$O_2_ (Aaron et al., 1992).

The work and O_2_ cost of breathing during exercise, and attendant perceived breathing difficulty, are minimized in young adults through several acute physiological adjustments. First, $\dot{\text{V}}$_E_ is maintained close to $\dot{\text{V}}$_A_ during exercise because of enhanced ventilation/perfusion ($\dot{\text{V}}$_A_/Q) relationships, while increased V_T_ reduces the “wasted” fraction of the breath [physiological dead space (V_D_)] (Johnson et al., 1994). Second, the behavior of the operating lung volumes is carefully controlled to minimize increases in elastic loading of the inspiratory muscles (Henke et al., 1988). Thus, in young adults, end-expiratory lung volume (EELV) reduction by expiratory muscle recruitment during exercise allows V_T_ expansion to about 50–60% of the vital capacity (VC) by encroachment on both the expiratory and the inspiratory reserve volumes (Henke et al., 1988). This helps mitigate the increased elastic work associated with breathing closer to total lung capacity (TLC). Finally, resistive work is minimized despite high flow rates during exercise by intra- and extra-thoracic airway dilatation (England and Bartlett, 1982; Warren et al., 1984).

**Figure 1:**
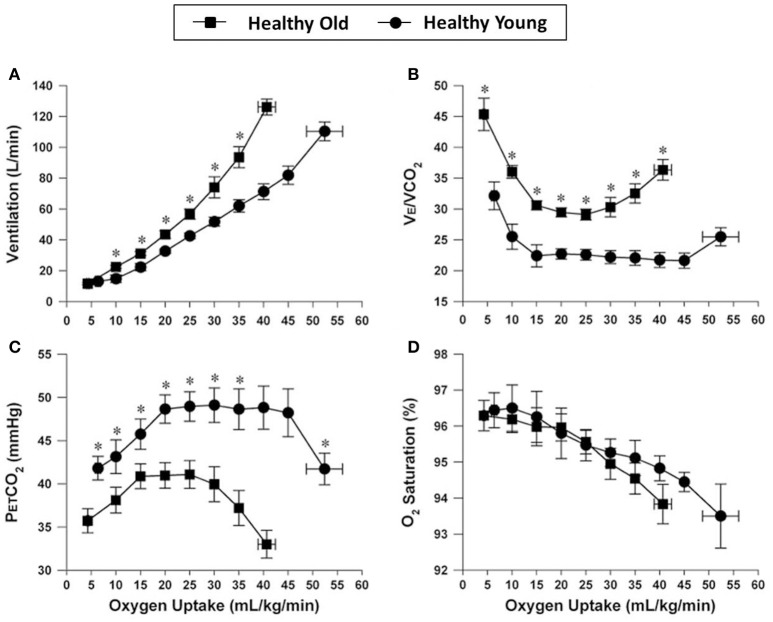
**Comparison of ventilation (A)**, ventilatory equivalent for carbon dioxide ($\dot{\text{V}}$_E_/$\dot{\text{V}}$CO_2_) **(B)**, end-tidal CO_2_ **(C)**, and arterial oxygen saturation **(D)**, all plotted against oxygen uptake, during incremental cycle exercise in healthy young and older adults. Values are mean ± SEM. ^*^P < 0.05 healthy young versus older adults. P_ET_CO_2_, partial pressure of end-tidal carbon dioxide; SpO2, oxygen saturation by pulse oximetry. Reproduced with permission from the publisher (Faisal et al., 2015).

These adaptations in pulmonary gas exchange and dynamic respiratory mechanics are variably attenuated with increasing age. These aging effects include progressive reductions in alveolar-capillary surface area for gas exchange, worsening of $\dot{\text{V}}$_A_/Q relationships, and smaller increases in V_T_ during exercise due to decreased chest wall compliance preventing normal declines in physiological dead space (Ofir et al., 2008b; Faisal et al., 2015). Submaximal $\dot{\text{V}}$_E_ is, therefore, increased at any given $\dot{\text{V}}$CO_2_, $\dot{\text{V}}$O_2_ or WR, reflecting the higher physiological dead space (Figure 1) (Faisal et al., 2015). The reduced efficiency in CO_2_ elimination also means that the central inspiratory drive to breathe is increased at any given WR compared with younger individuals (Dantzker and D'Alonzo, 1986; DeLorey and Babb, 1999; Prioux et al., 2000; Neder et al., 2003; Ofir et al., 2008b).

Age-related changes in the pulmonary connective tissue matrix are associated with reduced static elastic recoil pressure and driving pressure for expiratory flow (Frank et al., 1957; Olafsson and Hyatt, 1969; D'Errico et al., 1989; Johnson et al., 1994; Pride, 2005). Increased lung compliance and reduced airway tethering, together with changes in the autonomic balance of airway smooth muscle tone (possibly increased cholinergic influences), predisposes the elderly to expiratory flow limitation (EFL) (Turner et al., 1968; D'Errico et al., 1989; Johnson et al., 1991, 1994; Verbeken et al., 1992; Wilkie et al., 2012). During resting breathing, closing volume and the ratio of residual volume (RV) to TLC are both increased (Mittman et al., 1965; Anthonisen et al., 1969; Bode et al., 1976; McClaran et al., 1995). Resting IC is also diminished compared with younger individuals due to reduction in diaphragmatic strength with age, likely as a result of muscle atrophy and the age-related decrease in fast twitch fibers (Anthonisen et al., 1969; Tolep et al., 1995; Polkey et al., 1997). These changes are amplified by the higher ventilatory requirements of exercise, such that dynamic hyperinflation (DH); the transient increase of EELV above the resting value, can occur particularly at high $\dot{\text{V}}$_E_ in fit elderly individuals (DeLorey and Babb, 1999; Ofir et al., 2008b; Faisal et al., 2015).

Inability to reduce EELV in the elderly means that elastic work of breathing is increased and work sharing between expiratory and inspiratory muscles is compromised (Ofir et al., 2008b; Faisal et al., 2015). Moreover, the more rapid decline in dynamic IRV and earlier attainment of a plateau in the V_T_/$\dot{\text{V}}$_E_ relation have negative sensory consequences (DeLorey and Babb, 1999; Ofir et al., 2008b; Faisal et al., 2015). The combination of higher ventilatory demand and increased resistive and elastic loading of the respiratory muscles means that the O_2_ cost of breathing may represent as much as 13% of the total $\dot{\text{V}}$O_2_ in healthy older individuals (Harms et al., 1997). Generally, older more sedentary individuals avoid dyspnea provocation simply by avoiding high intensity exercise; however, critical respiratory mechanical constraints and attendant respiratory discomfort are well documented in older elite athletes who are determined to meet the challenge of exercising at high power outputs (Dempsey et al., 1984; Johnson et al., 1991). The age-related physiological derangements of pulmonary gas exchange and respiratory mechanics outlined above are exaggerated in patients in the early stages of various chronic lung diseases. This underlines the importance of using age-matched controls in studies of exercise pathophysiology in disease states.

### Cardiovascular responses to exercise in healthy elderly

Age-related declines in peak $\dot{\text{V}}$O_2_ are influenced by changes in the cardio-circulatory system, which undergoes significant structural changes during healthy aging (Arbab-Zadeh et al., 2004; Fujimoto et al., 2012; Strait and Lakatta, 2012). While global left ventricular (LV) systolic function and peak stroke volume (SV) are largely unchanged (Forman et al., 1992; Lakatta, 2003; Strait and Lakatta, 2012; Bhella et al., 2014), peak cardiac output falls by ~25% between 20 and 80 years of age (Fleg et al., 2005). Additionally, peak HR declines by 0.7 beats.min^−1^.year^−1^ (Tanaka et al., 2001) as a result of reduced beta(β)-adrenergic responsiveness with advancing age, which is partially offset by exercise-induced ventricular dilation (Filburn and Lakatta, 1984; Fleg et al., 1994; Brubaker and Kitzman, 2011). Thus, reduced HR and maldistributed cardiac output are responsible for the cardiac contribution to age-related decline in peak $\dot{\text{V}}$O_2_. Simply speaking, the cardiac response of the aging individual during exercise has been likened to that of a young person on β-blockers (Cheitlin, 2003).

Age attenuates the normal increases in HR, LV ejection fraction, and cardiac output observed during supine and upright incremental exercise (Geokas et al., 1990; Stratton et al., 1994). Aging hearts may utilize different mechanisms to increase SV during exercise compared to younger ones. Exercising elderly subjects try to maintain their SV via increases in end-diastolic volume, i.e., through the Frank-Starling mechanism; cardiac output may not be increased efficiently in subjects not exhibiting this cardiac dilatation. In younger subjects, SV is increased by a progressive decrease in end-systolic volume and little change in end-diastolic volume, in other words by an increase in the LV ejection fraction (Geokas et al., 1990; Stratton et al., 1994). It is worth noting that age does not alter the cardiac output–$\dot{\text{V}}$O_2_ relationship; however, for a given cardiac output, older subjects have decreased blood flow to the exercising leg muscles (Betik and Hepple, 2008).

The lactate threshold declines with age (Neder et al., 1999a; Pollock et al., 2015), though it increases with aging when expressed as % peak $\dot{\text{V}}$O_2_ (Iredale and Nimmo, 1997). Decreases in the lactate threshold occur less rapidly during aging than the observed decreases in $\dot{\text{V}}$O_2_ (Posner et al., 1987). These phenomena have been attributed to age-related decrease in maximal $\dot{\text{V}}$O_2_ (Reinhard et al., 1979; Cunningham et al., 1985; Posner et al., 1987). The occurrence of the lactate threshold at a higher percentage of maximum $\dot{\text{V}}$O_2_ in older individuals (Iredale and Nimmo, 1997) may also be attributed to decreased lactate production, improved clearance, or both. Moreover, the β-adrenergic receptor system has a significant effect on blood lactate concentration during exercise, with β-adrenergic stimulation increasing lactate production (Stainsby and Brooks, 1990). Thus, reduced β-adrenergic receptor sensitivity in advanced age may decrease lactate production, altering the balance between production and removal. Alteration in muscle fiber composition and recruitment may also play a role in circulating blood lactate levels (Spirduso, 1995).

## Chronic obstructive pulmonary disease

It is generally believed that smokers with unremarkable spirometric abnormalities, who are free of troublesome respiratory symptoms, need no treatment beyond the imperative of a smoking cessation intervention. However, this view is rapidly changing. Recent epidemiological studies have confirmed that activity-related dyspnea, activity restriction, poor quality of life, and increased risk of mortality are present in many symptomatic smokers with only minor spirometric abnormalities (Furlanetto et al., 2014; Regan et al., 2015; Woodruff et al., 2016). In this context, we now have a much better understanding of the heterogeneous nature of the physiological impairment in smokers with minor airway obstruction (Ofir et al., 2008a; Deesomchok et al., 2010; Chin et al., 2013; Guenette et al., 2014; Elbehairy et al., 2015a,b, 2016). Established abnormalities in mild COPD include: increased alveolar-to-arterial O_2_ tension gradient (A-aPO_2_) during resting breathing (Barbera et al., 1991; Rodriguez-Roisin et al., 2009; Elbehairy et al., 2015a); reduced diffusing capacity of the lungs for carbon monoxide (D_L_CO) and reduced transfer factor (Kirby et al., 2013; Harvey et al., 2015); increased peripheral airways resistance (Hogg et al., 2004; McDonough et al., 2011); maldistribution of alveolar ventilation (Buist, 1973); EFL, pulmonary gas trapping (increased ratio of RV to TLC) and reduced IC (Ofir et al., 2008a; Chin et al., 2013; Guenette et al., 2014; Elbehairy et al., 2015a,b).

Similar derangements of pulmonary gas exchange, dynamic respiratory mechanics and muscle function are seen in more advanced COPD. They differ from those changes observed in mild COPD in that they are more pronounced and occur at significantly lower $\dot{\text{V}}$_E_ and WR (O'Donnell et al., 2012; Neder et al., 2015; Faisal et al., 2016).

### Increased inspiratory neural drive during exercise in COPD: increased chemostimulation

The physiological adjustments that optimize pulmonary gas exchange and mechanics in youth and are attenuated by natural aging are further eroded by tobacco-related lung injury. Normally, the ventilatory response to exercise is coupled to metabolic demand (increasing $\dot{\text{V}}$CO_2_) throughout incremental exercise (i.e., $\dot{\text{V}}$_E_/$\dot{\text{V}}$CO_2_ = 1/[PaCO_2_ × (1 − V_D_/V_T_)]). In other words, the higher the $\dot{\text{V}}$_E_/$\dot{\text{V}}$CO_2_ (i.e., less “efficient” ventilation), the lower the level at which PaCO_2_ is regulated (i.e., CO_2_set-point) and the greater the fraction of the breath that is wasted as V_D_ (Wasserman et al., 1999). Poor ventilatory efficiency is a key physiological abnormality in symptomatic smokers with largely preserved forced expiratory volume in one second (FEV_1_) (Ofir et al., 2008a; Chin et al., 2013; Guenette et al., 2014; O'Donnell et al., 2014a; Neder et al., 2015; Elbehairy et al., 2015a,b). The physiological basis for this seems to stem from an enlarged V_D_ *per se*, rather than a small V_T_ or a low PaCO_2_ set point (Elbehairy et al., 2015a). In fact, added external V_D_ predictably increases $\dot{\text{V}}$_E_/$\dot{\text{V}}$CO_2_ in these patients (Chin et al., 2013). Additionally, reduced pulmonary perfusion and resultant areas of high $\dot{\text{V}}$_A_/Q mean that less CO_2_ is presented to the alveoli for removal with net dilution of PetCO_2_ from the expired V_T_ (Hansen et al., 2007; Elbehairy et al., 2015a). Regardless of the mechanism(s), the excessive ventilatory response erodes mechanical reserves, thereby contributing to exertional dyspnea and exercise intolerance (Ofir et al., 2008a; Chin et al., 2013; Guenette et al., 2014; Elbehairy et al., 2015a,b).

**Figure 2:**
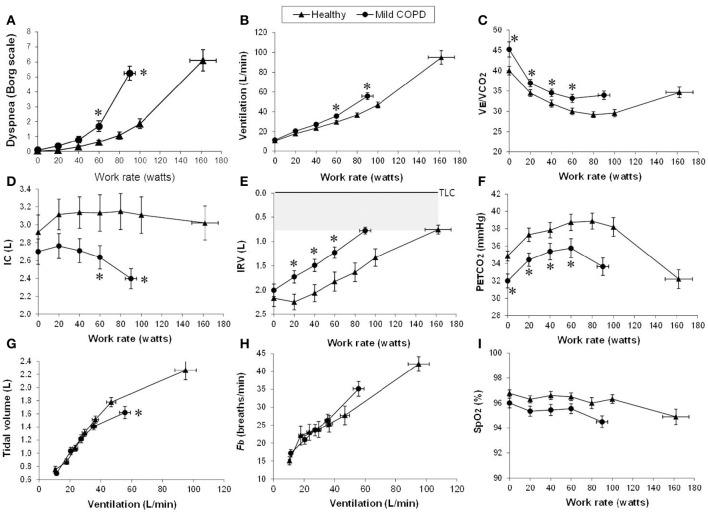
**Proposed panel displays for interpretation of key perceptual (A)**, ventilatory, and dynamic respiratory mechanical responses **(B–I)** to incremental exercise test in patients with chronic respiratory diseases. Data showing these responses in patients with mild COPD and age-matched healthy controls. Values are mean ± *SEM*. ^*^p < 0.05 mild COPD vs. healthy controls at rest, at standardized work rates or at peak exercise. V_E_/VCO_2_, ventilatory equivalent for carbon dioxide; IC, inspiratory capacity; IRV, inspiratory reserve volume; Fb, breathing frequency; PETCO_2_, partial pressure of end-tidal carbon dioxide; SpO_2_, oxygen saturation by pulse oximetry. Reproduced with permission from the publisher (Chin et al., 2013).

During the challenge of incremental exercise, the dominant abnormalities in mild COPD include (Figure 2): (1) increased chemostimulation of respiratory centers secondary to the effects of high physiological dead space compared with healthy controls, which is indirectly reflected by higher $\dot{\text{V}}$_E_/$\dot{\text{V}}$CO_2_ nadir and steeper $\dot{\text{V}}$_E_-$\dot{\text{V}}$CO_2_ slope; and (2) increased airways resistance and DH due to the combined effects of peripheral airway disease (EFL), increased ventilatory demand and central motor command output (Ofir et al., 2008a; Chin et al., 2013; Guenette et al., 2014; Elbehairy et al., 2015a,b). These combinations of increased mechanical loading of the muscles, dynamic functional muscle weakness (due to geometric muscle fiber shortening), and increased velocity of contraction means that efferent motor output to the respiratory muscles from cortical centers in the brain must increase to maintain adequate force generation (Pride and Macklem, 1986). Reduced IC during exercise (due to increased EELV) coupled with higher inspiratory neural drive (due to inefficient pulmonary gas exchange and increased mechanical loading) result in critical mechanical constraints and higher exertional dyspnea ratings earlier in exercise in mild COPD than in age-matched healthy controls (see also next section) (Ofir et al., 2008a; Chin et al., 2013; Guenette et al., 2014; Elbehairy et al., 2015a,b).

**Figure 3:**
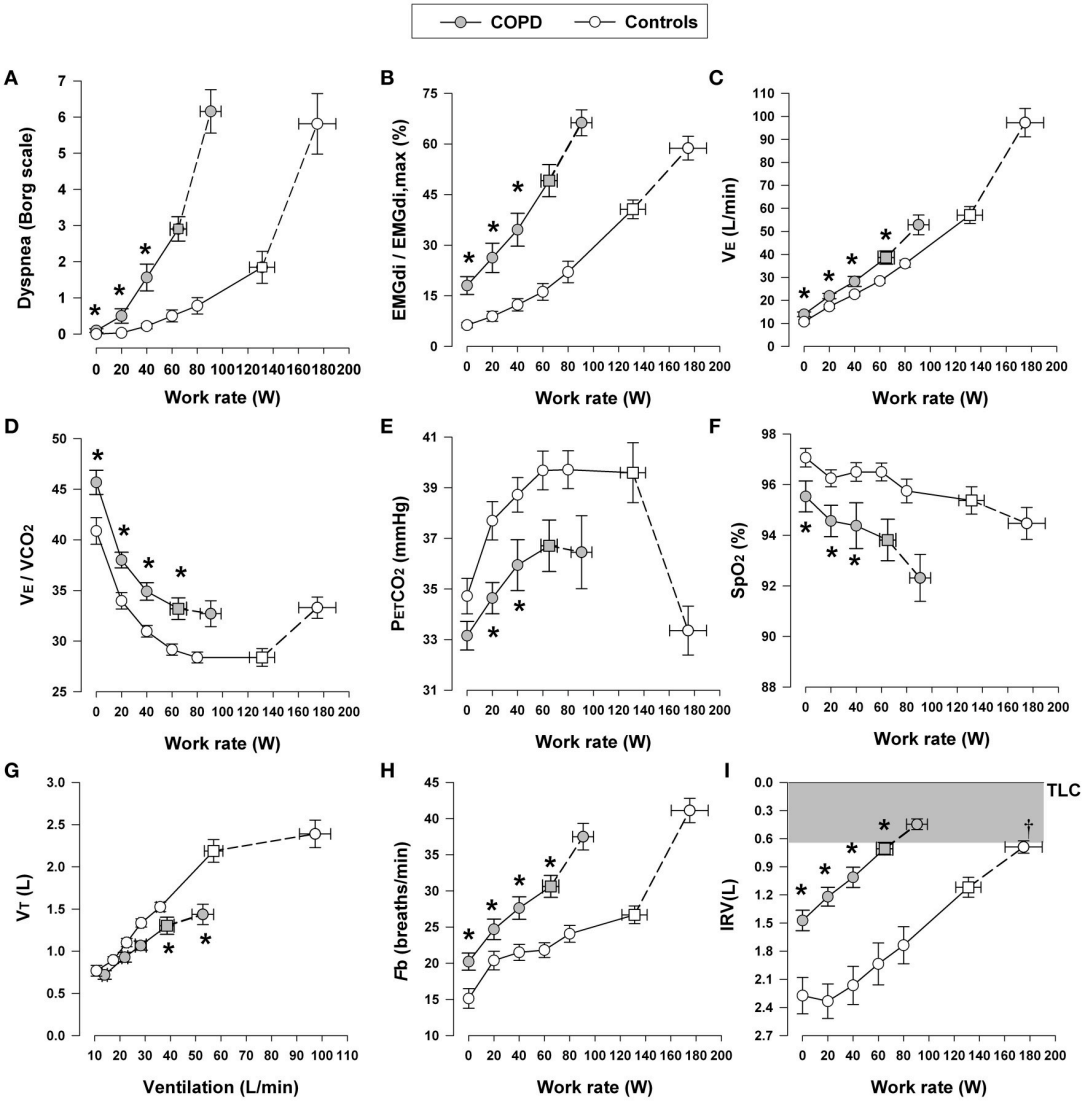
**Dyspnea intensity (Borg units) (A)**, diaphragm electromyography (EMGdi) **(B)** and selected ventilatory and indirect gas exchange responses **(C–I)** to incremental cycle exercise test in patients with moderate COPD and age-matched healthy controls. Values are mean ± *SEM*. Square symbols represent tidal volume-ventilation inflection points. ^*^p < 0.05 for COPD vs. control subjects at rest, at standardized work rates, at peak exercise, or at the tidal volume-ventilation inflection points. EMGdi/EMGdi,max, an index of inspiratory neural drive to the crural diaphragm; $\dot{\text{V}}$E, minute ventilation; V_E_/VCO_2_, ventilatory equivalent for carbon dioxide; PETCO_2_, partial pressure of end-tidal carbon dioxide; SpO_2_, oxygen saturation by pulse oximetry; VT, tidal volume; Fb, breathing frequency; IRV, inspiratory reserve volume; TLC, total lung capacity. Reproduced with permission from the publisher (Faisal et al., 2016).

In moderate-to-severe COPD, the progressively increasing intrinsic mechanical loading of the functionally weakened respiratory muscles requires augmented increases in efferent motor drive (from the motor cortex) to achieve a given force generation (Figure 3) (Gandevia et al., 1981; Gandevia, 1982; Turner, 1991; Faisal et al., 2016). Additionally, reflex stimulation of the central and peripheral chemoreceptors occurs as a result of: (1) $\dot{\text{V}}$_A_/Q abnormalities (decreased ventilatory efficiency, high $\dot{\text{V}}$_A_/Q lung units, and increased physiological dead space) (Caviedes et al., 2012; Neder et al., 2015); (2) critical arterial O_2_ desaturation (low $\dot{\text{V}}$_A_/Q lung units and reduced systemic mixed venous O_2_ in the blood) (Andrianopoulos et al., 2014; Moreira et al., 2014); and (3) increased acid-base disturbances (e.g., early metabolic acidosis) due to deconditioning or impaired cardiac function (Patessio et al., 1993; Pleguezuelos et al., 2016). In advanced COPD, alveolar hypoventilation with CO_2_ retention can occur, reflecting critical mechanical limitation and respiratory muscle dysfunction, particularly in the setting of high V_D_ and restricted V_T_ expansion (see below) (O'Donnell et al., 2002, 2014a). Finally, the negative haemodynamic consequences of combined resting and dynamic hyperinflation may reduce cardiac output, and thus O_2_ delivery to the contracting peripheral muscles, amplifying metabolic acidosis and ventilatory stimulation (Chiappa et al., 2008; Laveneziana et al., 2009, 2011; Vasilopoulou et al., 2012).

**Figure 4:**
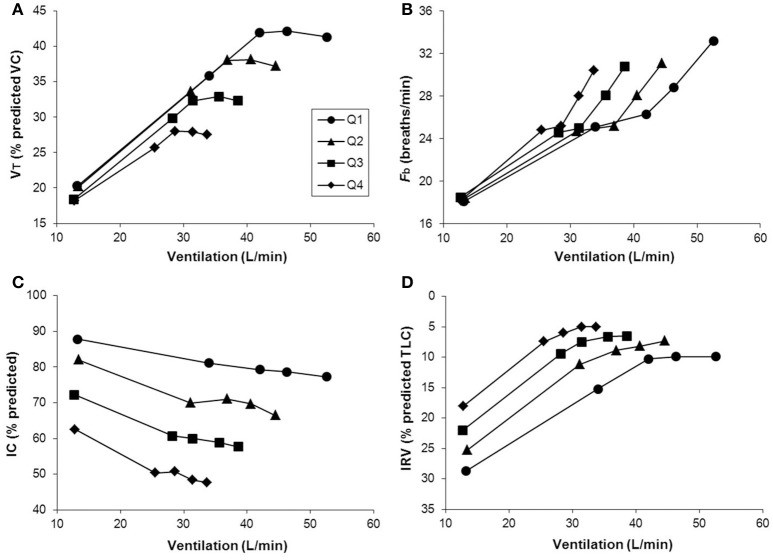
**Tidal volume (VT) (A)**, breathing frequency (Fb) **(B)**, dynamic inspiratory capacity (IC) **(C)**, and inspiratory reserve volume (IRV) **(D)** are shown plotted against minute ventilation ($\dot{\text{V}}$E) in four disease severity quartiles based on FEV1 %predicted during constant work rate exercise in patients with COPD. Note the clear inflection (plateau) in the VT/$\dot{\text{V}}$E relationship which coincides with a simultaneous inflection in the IRV. After this point, further increases in VE are accomplished by accelerating Fb. Data plotted are mean values at steady-state rest, isotime (i.e., 2, 4 min), the VT/$\dot{\text{V}}$E inflection point, and peak exercise. VC, vital capacity; TLC, total lung capacity. Reproduced with permission from the publisher (O'Donnell et al., 2012).

As previously reported, V_D_/V_T_ was found to be higher in mild COPD patients compared with healthy controls due to higher V_D_ rather than smaller V_T_ (Elbehairy et al., 2015a). Similar to heart failure (Woods et al., 2010), V_D_/V_T_ worsens in tandem with COPD severity (O'Donnell et al., 2014b). Interestingly, while the most commonly-used parameter of ventilatory efficiency ($\dot{\text{V}}$_E_-$\dot{\text{V}}$CO_2_ slope) increases from mild to severe heart failure (Sue, 2011); it decreases from mild to (very) severe COPD (Neder et al., 2015). This seemingly paradoxical finding is explained by the worsening mechanical constraints on increasing ventilation in COPD (Figure 4) (O'Donnell et al., 2012, 2014b), which in end-stage disease can lead to hypercapnia at end-exercise (O'Donnell et al., 2002; Poon et al., 2015). Thus, caution is necessary when using the $\dot{\text{V}}$_E_-$\dot{\text{V}}$CO_2_ relationship to interpret the trajectory of increases in physiological dead space in patients with limiting mechanical constraints.

### Abnormal dynamic respiratory mechanics during exercise in COPD

Increased respiratory motor drive and respiratory muscle effort occur in COPD due to increased elastic loading [including increased inspiratory threshold loading due to the effect of intrinsic positive end-expiratory pressure (PEEP)], decreased dynamic lung compliance, and increased resistive loading of the respiratory muscles (Potter et al., 1971; Dodd et al., 1984; Jolley et al., 2015; Faisal et al., 2016). IC is a useful non-invasive marker of dynamic respiratory mechanics in pulmonary diseases, as it indicates how close the patient is breathing to TLC. In both mild and advanced COPD, critical dynamic mechanical constraints are indicated by DH and by premature encroachment of end-inspiratory lung volume (EILV) on TLC, i.e., the attainment of a critically reduced IRV (Figures 2, 3) (Ofir et al., 2008a; O'Donnell et al., 2011, 2012; Chin et al., 2013; Guenette et al., 2013, 2014; Elbehairy et al., 2015a,b; Faisal et al., 2016). Thus, V_T_ becomes positioned close to TLC and the upper reaches of the S-shaped pressure-volume relation of the relaxed respiratory system, where compliance is decreased and the inspiratory muscles are functionally weakened. This explains the blunted V_T_ response and relative tachypnea in COPD compared with healthy controls (Figures 2, 3) (Chin et al., 2013; Faisal et al., 2016). This increased breathing frequency and velocity of shortening of the inspiratory muscles causes further functional weakness of those muscles (Leblanc et al., 1988).

Expiratory muscle activity is relatively increased in COPD, but fails to prevent DH (Ciavaglia et al., 2014; Laveneziana et al., 2014). In fact, excessive expiratory muscle recruitment may have deleterious hemodynamic effects, which further compromise exercise performance (Potter et al., 1971; Kyroussis et al., 2000). Evidence that respiratory muscle fatigue is present at the limits of tolerance in advanced COPD is inconclusive, but some degree of dynamic functional weakness of the overloaded inspiratory muscles is measurable in such patients (Mador et al., 2000); however, overt “static” (resting) inspiratory muscle weakness is reported in a subset of advanced COPD patients and is multifactorial (Gosselink et al., 1996; Charususin et al., 2016). In this group, it is anticipated that further dynamic respiratory muscle weakness will occur at higher ventilation during progressive exercise (Rodrigues et al., in press).

### Cardiovascular responses to exercise in COPD

Despite the fact that cardio-circulatory abnormalities are well documented in patients in the early stages of COPD, as tobacco smoking is a common risk factor (Sin et al., 2005; Malerba et al., 2011), crude non-invasive assessment of cardiac function during exercise may not be different from control subjects. For example, there is relatively greater variability in HR responses to incremental exercise in mild COPD patients compared with age-matched controls. HR at submaximal WR and $\dot{\text{V}}$O_2_ is often higher in patients with mild COPD compared with age-matched controls (Chin et al., 2013; Elbehairy et al., 2015a,b). However, this finding *alone* is not diagnostic for cardiac abnormality. On the other hand, O_2_ pulse (a surrogate for SV during exercise) and HR reserve (predicted maximal HR minus peak HR) were not different during exercise in a group of patients with mild-moderate COPD (FEV_1_ 62% predicted) and patients with more preserved FEV_1_ (94% predicted) compared with control subjects (Wang et al., 2011; Elbehairy et al., 2015a). Use of these crude exercise measures might not, therefore, be conclusive when assessing early cardiovascular changes in patients with mild COPD.

Patients with mild COPD are unlikely to develop pulmonary arterial hypertension (PAH) at rest, though one report suggests that it presented in up to 17% of their sample (Gupta et al., 2011). These patients would also have an increased likelihood of developing PAH during exercise, as supported by Portillo et al. who showed an abnormal increase of pulmonary artery pressure (PAP) during exercise in 70% of patients with GOLD grade 2 COPD (Portillo et al., 2015). Patients with exercise-induced PAH are also more prone to develop resting PAH in subsequent years (Kessler et al., 2001). To the best of our knowledge, there are no studies of exercise-induced PAH in GOLD grade 1 COPD patients.

The effect of more severe COPD on cardiac performance during exercise is complex, multifactorial, difficult to assess using HR responses during CPET and generally needs additional testing. Severe lung hyperinflation and excessive expiratory muscle recruitment can impair venous return and reduce right ventricular preload (Aliverti and Macklem, 2001). The large intrathoracic pressure swings generated during exercise to overcome increased elastic and resistive loads may result in LV dysfunction (increased LV afterload), especially in patients with cardiac comorbidity. Additionally, several studies have demonstrated increased pulmonary vascular resistance (PVR) during exercise in moderate-severe COPD (Light et al., 1984; Mahler et al., 1984; Magee et al., 1988; Oswald-Mammosser et al., 1991), which results from emphysematous vascular destruction in conjunction with reduced volume or compliance of the pulmonary vascular bed and, in some cases, from vasoconstriction due to regional alveolar hypoxemia (Light et al., 1984; Mahler et al., 1984; Magee et al., 1988; Agusti et al., 1990; Oswald-Mammosser et al., 1991). In some cases, mechanical compression of intra-alveolar vessels may also occur as a result of regional DH. Finally, severe lung hyperinflation can mechanically impede cardiac output (e.g., tamponade) in very advanced COPD (Stone et al., 2016; Watz, 2016).

PAP and right ventricular afterload are generally much higher in moderate-severe COPD than in health at a given cardiac output (Matthay et al., 1980; Agusti et al., 1990). Right ventricular afterload during exercise is increased in COPD because of the increased PVR associated with breathing at lung volumes close to TLC, and earlier studies showed failure to increase right ventricular ejection fraction despite a rise in right ventricular end-diastolic pressure (Matthay et al., 1980; Magee et al., 1988; Agusti et al., 1990; Vizza et al., 1998). LV ejection fraction is generally preserved in COPD in the absence of concomitant ischemic heart disease or hypertension (Morrison et al., 1987; Vizza et al., 1998); however, LV diastolic function may be impaired because of ventricular interdependence, i.e., increased tension or displacement of the right ventricle as a result of increased PVR, which may impede LV diastolic filling (Morrison et al., 1987; Vizza et al., 1998). Cardiac output has been found to increase normally with $\dot{\text{V}}$O_2_ during submaximal exercise in COPD, despite the increased PVR, but peak cardiac output (and $\dot{\text{V}}$O_2_) reaches lower values than in health (Mahler et al., 1984; Montes de Oca et al., 1996). This maintained cardiac output profile is attributable to decreased SV, and correspondingly increased HR, at a given $\dot{\text{V}}$O_2_ in COPD patients compared with healthy individuals (Mahler et al., 1984).

### Exertional dyspnea in COPD

**Figure 5:**
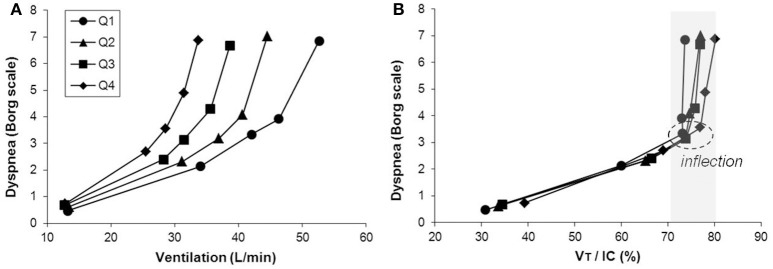
**Inter-relationships are shown between exertional dyspnea intensity, ventilation ($\dot{\text{V}}$E) (A)** and the VT/IC ratio **(B)** in four disease severity quartiles based on FEV1 %predicted during constant work rate exercise in COPD. After the VT/IC ratio plateaus (i.e., the VT inflection point), dyspnea rises steeply to intolerable levels. There is a progressive separation of dyspnea/V$\dot{\text{V}}$E plots with worsening quartile. Data plotted are mean values at steady-state rest, isotime (i.e., 2, 4 min), the VT/$\dot{\text{V}}$E inflection point, and peak exercise. IC, inspiratory capacity, VT, tidal volume. Reproduced with permission from the publisher (O'Donnell et al., 2012).

Exercise performance in COPD is primarily limited by ventilatory factors and accompanying intolerable respiratory discomfort in the majority of patients with more advanced COPD. Progressive reduction of resting IC (as resting lung hyperinflation increases) with disease progression helps explain the diminishing operating limits for V_T_ expansion and progressively earlier attainment of a minimal IRV during exercise (Figure 5) (O'Donnell et al., 2012). The point at which V_T_ reaches a critical minimal IRV is important during exercise. This is where the disparity between increasing inspiratory neural drive and the muscular/mechanical response of the respiratory system abruptly widens [i.e., where neuromechanical dissociation (NMD) begins] and marks the threshold beyond which dyspnea intensity rises sharply to intolerable levels (O'Donnell et al., 2004, 2006, 2012). The hypothesis that activity-related dyspnea and exercise intolerance are closely related to increased inspiratory neural drive and NMD in COPD is supported by studies showing that bronchodilator therapy, which improves dynamic mechanics (increases resting IC), delays mechanical limitation and partially restores neuromechanical coupling, delays the dyspnea threshold and prolongs exercise endurance time (O'Donnell et al., 2004, 2006). Additionally, interventions that directly or indirectly reduce inspiratory neural drive [e.g., supplemental oxygen (O'Donnell et al., 1997, 2001; Somfay et al., 2001), opiates (Mahler et al., 2009; Jensen et al., 2012; Johnson et al., 2013; Rocker et al., 2013; Ekström et al., 2015), and exercise training (Carrieri-Kohlman et al., 1996; Wadell et al., 2013)] can further improve dyspnea and exercise tolerance.

## Bronchial asthma

We focused in our review on chronic respiratory diseases in which functional consequences are largely non-reversible; we provide only a brief outline of the main pathophysiological mechanisms of exercise intolerance in patients with bronchial asthma. Asthma is a heterogeneous airway inflammatory disease characterized by EFL which is largely or fully reversed either spontaneously or after treatment (GINA, 2016). Patients with asthma respond variably to the stress of exercise depending on the degree of obstruction and bronchodilator reversibility. Furthermore, some atypical causes of exertional dyspnea are more common in asthma than other chronic respiratory diseases, including psychogenic hyperventilation (Ritz et al., 2008), dysfunctional breathing (Agache et al., 2012) and paradoxical vocal cord motion (Low et al., 2017). Thus, exercise responses in asthma characteristically vary among patients and in a given patient on different days (Del Giacco et al., 2015).

### Increased inspiratory neural drive during exercise in asthma: increased chemostimulation

The presence of recurrent bronchoconstriction (or persistent airflow obstruction) in some patients with bronchial asthma is associated with resting and exertional dyspnea which contributes to a sedentary lifestyle (Ford, 2003; Verlaet et al., 2013; Damera and Panettieri, 2014). Excessive exercise ventilation due to an increased chemostimulation (early metabolic acidosis) (Neder et al., 1999b) and increased wasted ventilation (Anderson et al., 1972) have been reported and might accelerate the rate of airway dehydration, a potent trigger for mediator release and exercise-induced bronchoconstriction in some patients (Parsons et al., 2013). Bronchoconstriction may cause an uneven distribution of ventilation relative to perfusion with consequent widening of the A-aPO_2_ (Wagner et al., 1987). However, most studies reported that, on average, asthmatic patients did not present with substantial hypoxemia or hypercapnia (Feisal and Fuleihan, 1979; Graff-Lonnevig et al., 1980; Wagner et al., 1987). Yet, an influential study (Haverkamp et al., 2005) reported that a sizeable fraction of habitually active asthmatic subjects (~35%) developed gas exchange abnormalities during exercise, which was associated with more extensive EFL, DH and increased airway resistance. Of note, post-exercise sputum histamine was correlated with measures of gas exchange inefficiency (Haverkamp et al., 2005). These data suggest that release of airway-related mediators worsened ventilation distribution during exercise (Haverkamp et al., 2005). In fact, a more pro-active treatment of airway inflammation with higher doses of inhaled steroids was associated with improved exertional A-aPO_2_ in mild to moderate asthma (Haverkamp et al., 2007). Thus, some patients with apparently well-controlled asthma may present with subtle physiological abnormalities which are amplified by the stress of exercise (Feisal and Fuleihan, 1979; Wagner et al., 1996; O'Donnell and Laveneziana, 2007; Rossman et al., 2014; Del Giacco et al., 2015). In patients with difficult to control asthma, CPET has been found useful to ascertain whether or not there is a “respiratory” cause of exercise limitation, a finding that impacts on disease management (McNicholl et al., 2011).

### Abnormal dynamic respiratory mechanics during exercise in asthma

Gas trapping and lung hyperinflation might occur in patients with bronchial asthma depending on the prevailing level of airway narrowing (Vermeulen et al., 2016). In this context, the sub-group of patients with “fixed,” poorly-reversible airflow obstruction may present with mechanical-ventilatory constraints and exertional dyspnea akin to COPD (O'Donnell and Laveneziana, 2007). At the other end of the functional spectrum, asthmatic adults (Rossman et al., 2014) and children (Santuz et al., 1997) with largely preserved FEV_1_ may present with mechanical and pulmonary gas exchange responses indistinguishable to sedentary controls. Interestingly, however, there is some evidence that patients with apparently-preserved lung function may develop significant inspiratory constraints secondary to higher operating lung volumes. As expected, these abnormalities were associated with lower exercise endurance and higher exertional dyspnea (Laveneziana et al., 2013b). These patients may present with tidal EFL leading to DH and a critically-low IRV (Kosmas et al., 2004). Similar to COPD, a V_T_ inflection point marked a change in dyspnea quality from “my breathing requires more effort” to “I cannot take a deep breath in,” thereby indicating that the inspiratory constraints are centrally perceived as an abnormal response (Kosmas et al., 2004; Laveneziana et al., 2013b; Vermeulen et al., 2016).

The reported variability in the presence and extent of mechanical and gas exchange abnormalities in asthma might be partially related to the complexities involved in the control of bronchomotor tone during exercise (Pellegrino et al., 1998; Crimi et al., 2002; Rossman et al., 2014). Thus, increases in operating lung volumes, if associated with increases in V_T_, are expected to dilate the airways due to the augmented radial traction (Pellegrino et al., 1998). In fact, airway caliber has been found to fluctuate inversely with decreased and increased work load in asthmatics (Crimi et al., 2002). These findings raised the hypothesis that stretching of airway smooth muscle (ASM) might convey an important influence on airway function during exercise in these patients (Rossman et al., 2014). Recent evidence, however, demonstrates that the influence of lung stretching on ASM contractile activity is dynamically modulated by other potent physiological mechanisms, including vagally-mediated neuromuscular reflexes, parasympathetic tone, sympathetic excitation/circulating catecholamines and release of airway-derived mediators (Klansky et al., 2016). Increases in EELV might also have some beneficial effects in increasing airway diameter and/or reducing airway closure, due to airways-lung parenchyma interdependence (Brown et al., 2001). These sources of variability would add to the large circadian fluctuations in airway inflammation (Durrington et al., 2014) and further increase the heterogeneity of exercise responses in individual patients over time (Del Giacco et al., 2015).

## Restrictive lung diseases

Restrictive lung disorders (e.g., lung parenchymal diseases, neuromuscular disorders, chest wall restriction, and pulmonary resection) are characterized by an inability to expand V_T_ appropriately during the increased metabolic demand of exercise. Here, we focus solely on the interstitial lung diseases (ILD). Exercise intolerance is multifactorial in ILD, but intolerable exertional symptoms, increased central inspiratory neural drive (relative to maximum), and abnormal cardiac function, in varying combinations, are important contributors (Krishnan and Marciniuk, 2002).

The pathophysiological hallmark of ILD is reduced static lung compliance (i.e., increased lung elastic recoil), which simultaneously restricts lung volume expansion and increases the driving pressure for expiratory airflow; thus, TLC, VC, and IC are reduced while the ratio of FEV_1_/FVC is usually increased (Parker et al., 2011). At rest, arterial blood gases may appear normal or reveal mild hypoxemia and a compensated respiratory alkalosis (American Thoracic Society, 2000). Disruption of the pulmonary microvasculature and the alveolar-capillary interface in ILD causes impaired gas exchange (i.e., decreased arterial O_2_ saturation, widened A-aPO_2_, and decreased D_L_CO) (American Thoracic Society, 2000; Egan et al., 2005; Holland et al., 2008; Parker et al., 2011; Cortes-Telles et al., 2014). At rest, the increased ventilatory demand secondary to increased $\dot{\text{V}}$_A_/Q abnormalities, coupled with increased elastic loading of the inspiratory muscles, can result in an increased work and O_2_ cost of breathing (American Thoracic Society, 2000; Faisal et al., 2016).

### Increased inspiratory neural drive during exercise in ILD: increased chemostimulation

**Figure 6:**
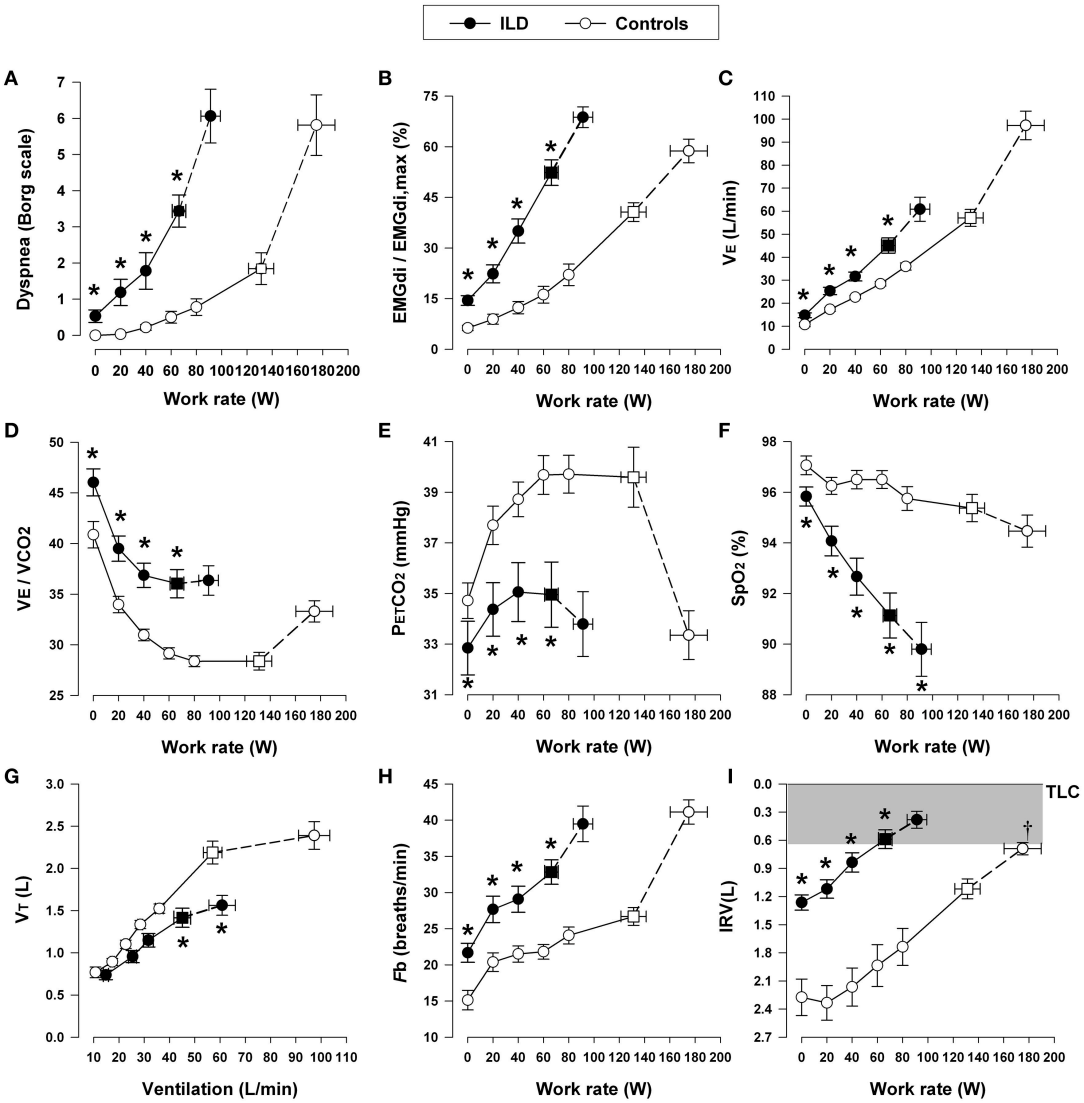
**Dyspnea intensity (Borg units) (A)**, diaphragm electromyography (EMGdi) **(B)**, and selected ventilatory and indirect gas exchange responses **(C–I)** to incremental cycle exercise test in patients with interstitial lung disease (ILD) and age-matched healthy controls. Values are mean ± *SEM*. Square symbols represent tidal volume-ventilation inflection points. ^*^p < 0.05 for ILD vs. control subjects at rest, at standardized work rates, at peak exercise, or at the tidal volume-ventilation inflection points. EMGdi/EMGdi,max, an index of inspiratory neural drive to the crural diaphragm; $\dot{\text{V}}$E, minute ventilation; $\dot{\text{V}}$E/$\dot{\text{V}}$CO_2_, ventilator equivalent for carbon dioxide; PETCO_2_, partial pressure of end-tidal carbon dioxide; SpO_2_, oxygen saturation by pulse oximetry; VT, tidal volume; Fb, breathing frequency; IRV, inspiratory reserve volume; TLC, total lung capacity. Reproduced with permission from the publisher (Faisal et al., 2016).

Similar to COPD, central inspiratory neural drive to breathe is higher when metabolic and ventilatory demand acutely increase during exercise in patients with ILD compared with healthy controls at any given work rate (Faisal et al., 2016) (Figure 6). This reflects (*in highly variable combinations*) the increased chemostimulation of central bulbo-pontine respiratory control centers and the increased efferent (cortical) motor output as a result of increased elastic loading of the respiratory muscles (Nishimura et al., 1989).

As discussed in COPD, increased chemostimulation in ILD similarly results from the effects of high $\dot{\text{V}}$_E_/$\dot{\text{V}}$CO_2_: reduced efficiency of CO_2_ elimination occurs as a result of increased wasted ventilation and/or relative alveolar hyperventilation due to changes in the CO_2_ set-point (Figure 6) (O'Donnell et al., 2007; Faisal et al., 2016). A key finding in fibrosing ILD is arterial hypoxemia with widened A-aPO_2_ during exercise (Hamer, 1964; Weitzenblum et al., 1983; Agusti et al., 1991; Hughes et al., 1991), which can occur in early stages of the disease, even before resting pulmonary function tests show overt impairment in D_L_CO and lung mechanics (Johnson et al., 1960; Keogh et al., 1984).

The mechanisms of arterial O_2_ desaturation with exercise include: inter- and intra-regional $\dot{\text{V}}$_A_/Q inequalities in the lungs, increased perfusion of units with low $\dot{\text{V}}$_A_/Q with poorly oxygenated mixed venous blood, diffusion disequilibrium with decreased pulmonary capillary transit time, and, in some individuals, increased intra-cardiac and intra-pulmonary right-to-left shunting (Hamer, 1964; Weitzenblum et al., 1983; Agusti et al., 1991; Hughes et al., 1991). Alveolar hyperventilation in response to hypoxemia is associated with low PaCO_2_ with a concomitant increase in PAO_2_, which further contributes to widen exertional A-aPO_2_ (Risk et al., 1984). Alveolar hypoventilation is not commonly reported during exercise, even in advanced ILD, but severe V_T_ restriction in the setting of a fixed high V_D_ can potentially cause CO_2_ retention in end-stage disease (Javaheri and Sicilian, 1992). Correlations have been found between the low resting D_L_CO and arterial hypoxemia during exercise (Agusti et al., 1991), but there is considerable overlap in this relationship, particularly in patients with mild to moderate disease.

Additional sources of ventilatory stimulation in ILD may include: altered reflex afferent activation of vagal receptors in the lung parenchyma and airways (Paintal, 1969), early metabolic acidosis due to deconditioning, and increased peripheral muscle ergo-receptor activation (Hansen and Wasserman, 1996). As in COPD, additional ventilatory stimulation may arise in some individuals due to comorbidities or complications, such as obesity (i.e., increased metabolic loading), PAH, emphysema, and cardio-circulatory disease (American Thoracic Society, 2000; Parker et al., 2011; Faisal et al., 2016).

### Abnormal dynamic respiratory mechanics during exercise in ILD

During exercise, patients with ILD experience dynamic restrictive mechanical constraints, which, as already seen in COPD, are reflected in high V_T_/IC ratios and an early plateau of the V_T_ response as it reaches the critical minimal IRV earlier in exercise compared with healthy individuals (Figure 6) (Faisal et al., 2016). As in COPD, low V_T_ precludes a normal decrement in V_D_/V_T_ ratio, worsening ventilatory inefficiency and increasing inspiratory neural drive. This leaves tachypnea as the only available option to respond to the higher drive (Javaheri and Sicilian, 1992). This rapid and shallow pattern of breathing helps attenuate the effects of increased elastic work of breathing and the attendant respiratory discomfort; however, as ventilation increases during exercise, the work of breathing increases dramatically in order to overcome the high elastic loads of the stiff lungs and chest wall while breathing close to the reduced TLC (O'Donnell et al., 2000). Moreover, the increased velocity of shortening of the inspiratory muscles results in dynamic functional inspiratory muscle weakness. Consequently, in most instances, exercise intolerance in ILD is explained by true ventilatory limitation and associated severe dyspnea (Kabitz et al., 2006; Holland, 2010; Walterspacher et al., 2013; Panagiotou et al., 2016).

In ILD, the pressure-volume relationship of the entire respiratory system is contracted along its volume axis, but retains its S-shape. The resting IC and IRV are usually diminished. With exercise, EILV encroaches further on the upper non-linear extreme of the pressure-volume relationship “beyond the S-bend,” where there is significant elastic loading (Parker et al., 2011). V_T_ reaches a plateau at 50–60% of the reduced VC (or ~ 70% of IC) early in exercise: minimal dynamic IRV and the V_T_ plateau are reached together with a step increase in breathing frequency (Faisal et al., 2016). A few studies report that IC remains largely unaltered throughout exercise, reflecting a diminished expiratory reserve volume (ERV) and a reduced ability to decrease EELV (Marciniuk et al., 1994; O'Donnell et al., 1998). However, EFL has been described in some patients with ILD and may reflect co-existent airway obstruction as a result of smoking or actual airway involvement as part of the interstitial disease process (e.g., hypersensitivity pneumonitis) (Jones and Rebuck, 1979; Marciniuk et al., 1994). Interestingly, the presence of EFL in ILD was associated with worsening dyspnea compared with those with ILD who had normal airway function (Marciniuk et al., 1994).

Inspiratory muscle function is often relatively preserved in patients with ILD, reflecting the training effects of intrinsic mechanical loading and the mechanical advantage of the inspiratory muscles at the lower than normal operating lung volumes (DeTroyer and Yernault, 1980; O'Donnell et al., 1998). However, in some individuals, involvement of these muscles in the underlying systemic inflammatory disease process, the effects of cachexia, high dose oral steroids, malnutrition, electrolytic disturbances, and/or global skeletal muscle deconditioning may have a deleterious impact on function (Baydur et al., 2001; Kabitz et al., 2006; Panagiotou et al., 2016).

### Cardiovascular responses to exercise in ILD

The characteristic cardiac abnormality in ILD is increased PVR with consequent right ventricular hypertrophy ultimately leading to *corpulmonale* during the terminal phase of the illness (Weitzenblum et al., 1983; Sturani et al., 1986). LV ejection fraction and systolic pressures are usually preserved, as are pulmonary artery occlusion pressures (Lupi-Herrera et al., 1985; Bush and Busst, 1988). Cardiac output is usually normal at rest and during low levels of exercise in ILD, but the rate of rise of cardiac output is diminished at higher work rates, due in part to increased PVR (Lupi-Herrera et al., 1985; Bush and Busst, 1988). PAP is high at rest and further increases during exercise in the majority of patients with ILD (Hawrylkiewicz et al., 1982; Weitzenblum et al., 1983; Jezek et al., 1985). Values for PAP of ~ 40 mmHg are not unusual, even in moderate ILD during minimal activity. High levels of PAP are required during exercise to maintain cardiac output when PVR is increased; PAP is often double the normal value or higher (Weitzenblum et al., 1983; Sturani et al., 1986). Obliteration of the vascular bed by progressive parenchymal fibrosis is the main explanation for the reduced vascular bed and the increased PVR in ILD (Enson et al., 1975). Other factors contributing to the increased PVR are hypoxic vasoconstriction and reduced lung volume.

HR responses to incremental exercise in ILD are variable. HR at submaximal work rates is often higher than normal (Widimsky et al., 1977; Baughman et al., 1984), reflecting the relatively reduced SV and greater sympathetic stimulation secondary to hypoxemia. Maximal HR, however, is generally diminished, and there is adequate cardiac reserve at exercise termination (Faisal et al., 2016). Diminished cardiac reserve may become evident in patients with cardiac involvement in the disease process (i.e., sarcoidosis, Eklund et al., 1989) or in patients with additional extensive pulmonary vascular disease (i.e., scleroderma, Guttadauria et al., 1979). As mentioned in the COPD section, traditional assessments of cardiac function during exercise based mainly on HR measurement are often relatively insensitive.

### Exertional dyspnea in ILD

**Figure 7:**
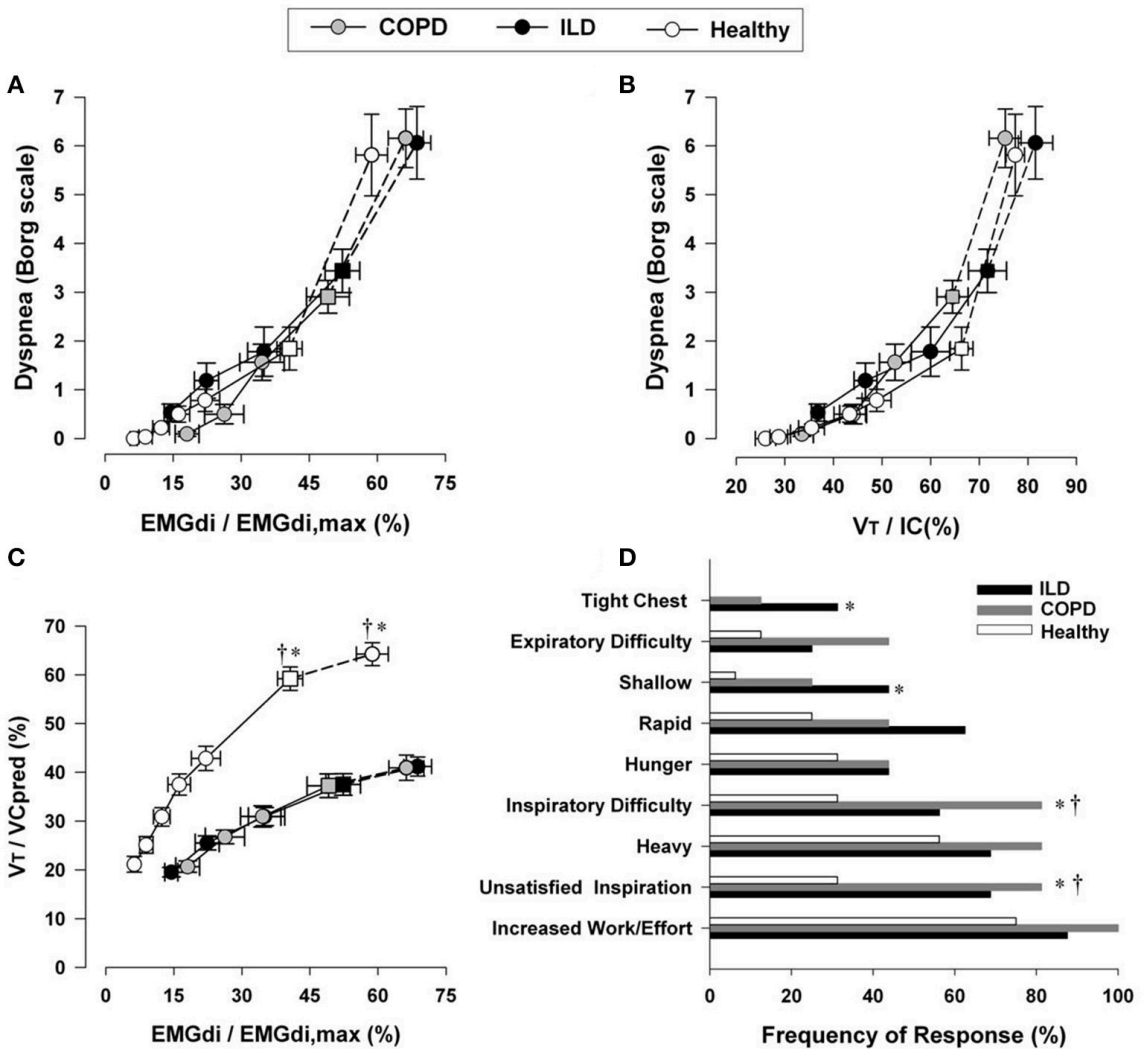
**Relation between dyspnea intensity (Borg units) and diaphragm electromyography (EMGdi) (A)** and V_T_/IC **(B)** during incremental cycle exercise test in patients with moderate COPD, ILD and age-matched healthy controls. **(C)** Shows the relation between V_T_/VC and EMGdi/EMGdi,max (an index of inspiratory neural drive to the crural diaphragm); note the similar blunted V_T_/VC response to the increased neural drive in both ILD and COPD patients compared with healthy subjects. Values are mean ± SEM. Square symbols represent tidal volume-ventilation inflection points. Selection frequency of descriptors of exertional dyspnea at end-exercise in the three groups is shown in **(D)**. ^*^p < 0.05 for ILD vs. control subjects and ^†^*p* < 0.05 for COPD vs. control subjects. COPD, chronic obstructive pulmonary disease; IC, inspiratory capacity; V_T_, tidal volume; VC, vital capacity; ILD, interstitial lung disease. Reproduced from with permission from the publisher (Faisal et al., 2016).

Increasing dyspnea intensity during CPET correlates well with increasing amplitude of the inspiratory neural drive to the diaphragm, the increasing esophageal pressure relative to maximum, and the increasing V_T_/IC ratio (a measure of prevailing mechanical constraints) in ILD (Figure 7) (O'Donnell et al., 1998; Faisal et al., 2016). Thus, as in COPD, dyspnea rises as a function of the increasing fractional inspiratory neural drive to the inspiratory muscles, increased contractile respiratory muscle effort, and intrinsic restriction of appropriate V_T_ expansion (Faisal et al., 2016). Similarly, interventions that attenuate chemostimulation, for example by delaying the rise in metabolic $\dot{\text{V}}$CO_2_ (e.g., exercise training, Ferreira et al., 2009; Dowman et al., 2013, 2014) or by reducing efferent output from respiratory centers (e.g., O_2_ supplementation, opiates), alleviate exertional dyspnea in patients with ILD (Visca et al., 2011; Bajwah et al., 2013; O'Donnell et al., 2016).

## Pulmonary arterial hypertension (PAH)

**Figure 8:**
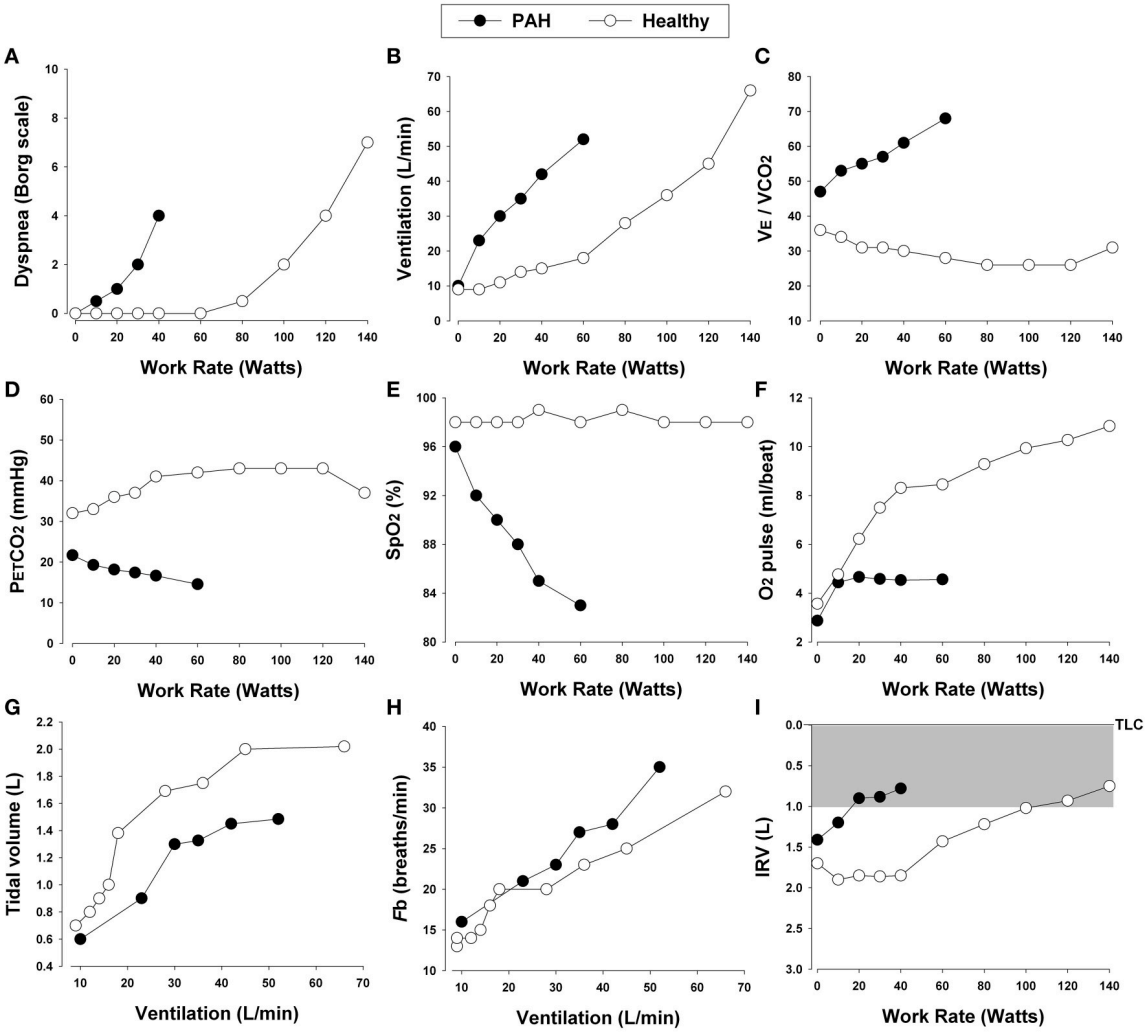
**Proposed panel displays for interpretation of key perceptual (A)**, ventilatory, and dynamic respiratory mechanical **(B–I)** responses to incremental exercise test in patients with chronic respiratory diseases. Data showing these responses in a patient with pulmonary arterial hypertension (PAH) and an age and gender matched healthy control. $\dot{\text{V}}$E/$\dot{\text{V}}$CO_2_, ventilatory equivalent for carbon dioxide; PETCO_2_, partial pressure of end-tidal carbon dioxide; SpO_2_, oxygen saturation by pulse oximetry; Fb, breathing frequency; IRV, inspiratory reserve volume; TLC, total lung capacity.

**Figure 9:**
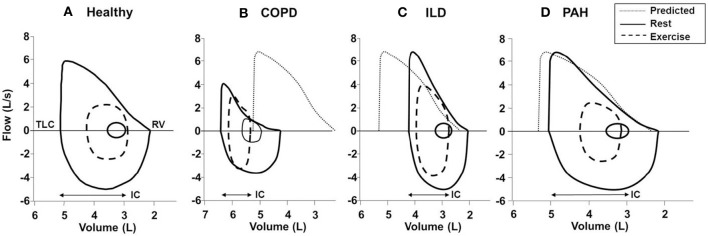
**Typical flow-volume curves in (A):** a healthy subject and patients with **(B)** COPD, **(C)** ILD, and **(D)** PAH. In the patient with COPD, there is a leftward shift of the curve with noticeable expiratory flow limitation during exercise (i.e., tidal loops at peak exercise exceed maximal expiratory envelope). In the patient with ILD, there is a rightward shift of the curve with no expiratory flow limitation and adequate reserves of inspiratory and expiratory flow at end exercise. Note the markedly reduced inspiratory reserve volume (IRV) in both ILD and COPD patients compared with healthy subject (IRV, TLC-end-inspiratory lung volume). In PAH; the flow-volume curve is close to the healthy subject due to absence of respiratory mechanical problem in most classical cases. Solid lines, maximal and tidal loops at rest; dashed lines, tidal loops at peak exercise; dotted lines, predicted normal maximal expiratory loop. COPD, chronic obstructive pulmonary disease; ILD, interstitial lung disease; TLC, total lung capacity; RV, residual volume; IC, inspiratory capacity; PAH, pulmonary arterial hypertension. Reproduced with permission from the publisher (O'Donnell et al., 1997, 1998).

Pulmonary hypertension is defined as a mean pulmonary artery pressure (mPAP) ≥25 mmHg at rest as measured through right heart catheterization (Hoeper et al., 2013). Although still controversial, values >30 mmHg during exercise are also deemed indicative of pulmonary hypertension (Hoeper, 2009). Pulmonary hypertension is consistently associated with reduced exercise capacity (Miyamoto et al., 2000; Riley et al., 2000a; Sun et al., 2001; Ferrazza et al., 2009). It has long been known that peak $\dot{\text{V}}$O_2_ and poor ventilatory efficiency correlate significantly with morbidity and mortality in patients with pulmonary hypertension (Schwaiblmair et al., 2012). In this review, we will focus primarily on cardiopulmonary response patterns in patients with increased PVR and PAP in whom dynamic ventilatory mechanics are largely preserved during exercise [e.g., idiopathic PAH (class 1) and chronic thromboembolic pulmonary hypertension (class 4)]. We will therefore exclude consideration of patients with pulmonary hypertension secondary to primary cardiac diseases (class 2) or COPD or restrictive lung diseases (class 3). Typical CPET findings in patients with PAH include (Figure 8): (1) inefficient intra-pulmonary gas exchange; (2) variable degrees of O_2_ desaturation; (3) hyperdynamic circulatory and ventilatory responses; (4) reduced peak $\dot{\text{V}}$O_2_ and/or impaired rate of submaximal $\dot{\text{V}}$O_2_; and (5) variable combinations of leg effort and dyspnea as the limiting symptoms. Thus, PAH is usually suspected when ventilatory responses to exercise and the inspiratory neural drive to breathe are abnormally elevated despite a relatively preserved maximal flow-volume loop (Figure 9) (Theodore et al., 1986; Reybrouck et al., 1998; Miyamoto et al., 2000; Riley et al., 2000a; Sun et al., 2001; Wensel et al., 2002).

### Increased inspiratory neural drive during exercise in PAH: increased chemostimulation

In patients with PAH, $\dot{\text{V}}$_E_ at any given submaximal exercise intensity is increased, reflecting poor pulmonary perfusion relative to ventilation (larger wasted ventilation) and, in some patients, alveolar hyperventilation (low CO_2_ set-point) (Theodore et al., 1986; Reybrouck et al., 1998; Sun et al., 2001; Wensel et al., 2002). As in COPD and ILD, in PAH, high V_D_/V_T_ is related not only to increased areas of high $\dot{\text{V}}$_A_/Q but also to a low V_T_ (see below). Thus, patients with PAH present with poor ventilatory efficiency expressed either as a high $\dot{\text{V}}$_E_/$\dot{\text{V}}$CO_2_ nadir or a steep $\dot{\text{V}}$_E_-$\dot{\text{V}}$CO_2_ slope (Figure 8) (Reybrouck et al., 1998; Mitani et al., 2002; Schwaiblmair et al., 2012). In most patients, increased dyspnea scores at relatively low levels of exercise are commensurate with the exaggerated increases in ventilation. Thus, increased neural drive secondary to increased chemostimulation is likely a key mechanism of increased dyspnea intensity in PAH relative to healthy controls.

The $\dot{\text{V}}$_E_-$\dot{\text{V}}$CO_2_slope and $\dot{\text{V}}$_E_/$\dot{\text{V}}$CO_2_ nadir may further increase if another source of ventilatory stimulus adds to the already-increased inspiratory neural drive. As discussed below, the ventilatory threshold is often lower in PAH than in health, reflecting reduced O_2_ delivery (due to cardiac impairment) to the active peripheral muscles and a greater reliance on anaerobic glycolysis (Riley et al., 2000b). Significant skeletal muscle deconditioning can arise as a result of dyspnea-related inactivity in patients with PAH, which again may be associated with an earlier metabolic acidosis and increased ventilatory stimulation (de Jesus Perez, 2014).

Arterial O_2_ desaturations with widening of the A-aPO_2_ typically occur to a variable extent in patients with established PAH (Riley et al., 2000a; Sun et al., 2001; Wensel et al., 2002). The widened A-aPO_2_ reflects critical $\dot{\text{V}}$_A_/Q inequalities and a diffusion defect as a result of reduced red cell transit times through the abnormal pulmonary vasculature (Riley et al., 2000a; Sun et al., 2001; Wensel et al., 2002). Cardiac impairment in PAH results in a lower mixed venous O_2_ saturation, which in the setting of alveolar units with low $\dot{\text{V}}$_A_/Q ratios negatively impacts arterial oxygenation (Hoeper et al., 2007). Any concomitant hypoxemia secondary to areas of low $\dot{\text{V}}$_A_/Q or right-to-left shunt (e.g., patent *foramen ovale*) may further increase ventilation and compromise O_2_ delivery (Steenhuis et al., 2000; Hoeper et al., 2007). PetCO_2_ levels are often diminished at higher levels of exercise compared with health, reflecting relative alveolar hyperventilation and dilution of expired CO_2_ (*see the COPD section*) (Riley et al., 2000b; Yasunobu et al., 2005; Hansen et al., 2007). In fact, it has been suggested that PAH should be suspected in patients who present with unexplained dyspnea and exercise limitation whose PetCO_2_ at the ventilatory threshold is <30 mmHg (Yasunobu et al., 2005).

### Abnormal dynamic respiratory mechanics during exercise in PAH

One would anticipate that dynamic ventilatory mechanics during exercise should not be abnormal in patients with PAH in the absence of concomitant COPD or restrictive thoracic diseases. However, it is conceivable that in some PAH patients with high ventilatory demand and rapid breathing frequency, DH may occur, particularly toward the end of exercise in the setting of EFL. Indeed, recent studies have provided evidence that EFL, DH, and restrictive mechanical constraints on V_T_ expansion can occur in a proportion of non-smokers with PAH with no evidence of EFL at rest (Laveneziana et al., 2013a; Richter et al., 2013; Laveneziana et al., 2015). As expected, these patients might present with increased dyspnea intensity at any given ventilation.

In general, and perhaps surprisingly, breathing pattern responses to exercise are usually more rapid and shallow in PAH than in health (Laveneziana et al., 2013a). In many patients, reduced V_T_ expansion is not easily explained by restrictive mechanics, as IRV is generally preserved or increased at peak exercise compared with healthy controls. Relative tachypnea may be related to activation of unmyelinated pulmonary C fibers and/or altered mechanoreceptor inputs from the right heart and pulmonary vasculature in a manner that remains incompletely understood (Aguggini et al., 1987). Additionally, studies have indicated that some patients with primary pulmonary hypertension may show a mild restrictive spirometric pattern as indicated by a modest reduction in TLC with preservation of the FEV_1_/FVC ratio (Horn et al., 1983). The underlying mechanism(s) remain unclear, but reduced lung compliance and dynamic inspiratory respiratory muscle weakness have been suggested as potential contributors (Phipps et al., 1983; Rich et al., 1987; Polkey et al., 1995; Sun et al., 2003; Deboeck et al., 2004; Escribano et al., 2005; Meyer et al., 2005). Regardless of the mechanism(s), a shallow breathing pattern will amplify the effect of a high V_D_ on the V_D_/V_T_ ratio and augment the increase in the ventilatory drive.

### Cardiovascular responses to exercise in PAH

A healthy pulmonary vasculature is a prerequisite for accommodating increased cardiac output during exercise while minimizing increases in PVR. In PAH, high PAP (secondary to the failure to recruit additional pulmonary vessels) and right ventricular pressure overload (with potential leftward shift of the interventricular septum) combine to impair the increase in SV during exercise (Janicki, 1990; Holverda et al., 2006). Thus, despite a compensatory increase in HR due to sympathetic over-excitation, cardiac output may fail to adequately meet the higher muscular demands for O_2_ (Janicki, 1990; Holverda et al., 2006).

Impairments in peripheral O_2_ delivery to skeletal muscles usually worsen as the demand for O_2_ increases, i.e., as exercise becomes more intense. Consequently, patients with PAH may present with a reduced slope of the relationship between $\dot{\text{V}}$O_2_ and WR (Δ$\dot{\text{V}}$O_2_/ΔWR <8–9 ml.min^−1^.watts^−1^) (Deboeck et al., 2004; Yasunobu et al., 2005). In patients with advanced PAH, Δ$\dot{\text{V}}$O_2_/ΔWR plateaus or even decreases near the end of exercise. The final consequence is a very low peak $\dot{\text{V}}$O_2_ due to the combination of low peak WR and decreased change in $\dot{\text{V}}$O_2_ for a given change in WR. Impairment in O_2_ delivery may also increase the contribution of anaerobic metabolism in the earlier stages of exercise leading to greater increases in CO_2_ production (from lactate buffering) for a given change in $\dot{\text{V}}$O_2_, i.e., an early lactate threshold (Deboeck et al., 2004; Yasunobu et al., 2005).

The impact of the tachycardic response on the ΔHR/Δ$\dot{\text{V}}$O_2_ relationship (O_2_ pulse, i.e., the amount of O_2_ taken in for a given heart beat) depends on the ability of active skeletal muscle to extract O_2_ from arterial blood and widened arterial-venous O_2_ difference (Wasserman et al., 1999). As most of the increase in the arterial-venous O_2_ difference occurs during early exercise, the HR/$\dot{\text{V}}$O_2_ ratio during late exercise is chiefly influenced by SV. Consequently, patients with PAH and impaired SV show a flattened, downward-displaced O_2_ pulse profile (Janicki, 1990; Wasserman et al., 1999; Deboeck et al., 2004; Yasunobu et al., 2005; Holverda et al., 2006). In advanced disease, a flat O_2_ pulse indicates a severely-impaired SV. The relative tachycardia that remains during the recovery period, observed as a slowed post-exercise HR decrease, has been found to be an ominous sign in PAH (Riley et al., 2000b; Deboeck et al., 2004; Ramos et al., 2012).

### Exertional dyspnea in PAH

The mechanisms of exertional dyspnea in PAH are less well studied than in the other chronic respiratory diseases. As in COPD and ILD, existing data support an important role of increased inspiratory neural drive, at least as indirectly assessed by measures of ventilatory output. The increased drive mainly reflects increased chemostimulation as a result of $\dot{\text{V}}$/Q abnormalities. In PAH, operating lung volumes are close to normal but DH has also been described in some individuals at higher exercise intensities (Laveneziana et al., 2013a; Richter et al., 2013; Laveneziana et al., 2015). Regardless of the mechanism of restriction, the inability to expand V_T_ in response to the increasing inspiratory neural drive (or inspired effort) during exercise contributes importantly to low peak ventilatory capacity and perceived respiratory discomfort. Traditionally, it has also been assumed that direct afferent from the right ventricle and pulmonary arterial afferents also play a contributory role, but the data to support this are inconclusive (Aguggini et al., 1987). It remains to be determined if neuro-mechanical dissociation occurs in PAH at the limits of tolerance, and whether qualitative descriptor choices are different from those of COPD and ILD.

## Comparison of exercise responses in COPD, ILD and PAH

At first glance, the four lung diseases under consideration are remarkably different in their underlying pathology, static respiratory mechanics, nature and extent of the mechanical load, respiratory muscle characteristics and recruitment patterns, and pulmonary gas exchange. A comparison of the tidal and maximal flow-volume loops during exercise allows easy diagnostic differentiation (Figure 9). However, differences in respiratory mechanics, inspiratory neural drive to the diaphragm, ventilation, breathing pattern and the behavior of dynamic IRV during conventional CPET are remarkably similar in obstructive and restrictive diseases when compared to the healthy condition. In COPD, V_T_ is restricted by the effects of resting and dynamic lung hyperinflation, whereas in ILD, the restriction is reflecting the reduced TLC and IRV (Faisal et al., 2016) (Figures 3, 6).

Patients with ILD often have better preservation of inspiratory muscle force-generating capacity, reflecting the mechanical advantage of lower operating lung volumes (Faisal et al., 2016). Furthermore, patients with ILD, unlike COPD patients, do not usually have to contend with inspiratory threshold and resistive loading. However, clinically stable ILD patients generally have relatively greater tachypnea and often more severe gas exchange abnormalities during exercise (O'Donnell et al., 2016). These latter abnormalities generally occur earlier in ILD than COPD and may precede the development of other resting pulmonary function tests abnormalities.

### Common mechanisms of dyspnea

In COPD and ILD, dyspnea during exercise fundamentally reflects an imbalance between the increased demand to breathe and the ability to meet that demand (Scano et al., 2010). The rise in dyspnea intensity correlates closely with the following physiological ratios: $\dot{\text{V}}$_E_/MVC (maximum ventilatory capacity); oesophageal pressure (Pes)/Pes_max_; V_T_/IC or EILV/TLC; and inspiratory neural drive to the diaphragm relative to maximum (EMGdi/EMGdi_max_) (Figure 7) (Gandevia and Hugh-Jones, 1957; Leblanc et al., 1988; Laveneziana et al., 2013a; Guenette et al., 2014; Elbehairy et al., 2016; Faisal et al., 2016). This indicates that respiratory discomfort is provoked when there is critical encroachment on reserves of ventilatory output, muscle force generation, V_T_ expansion or inspiratory neural drive to the diaphragm (Gandevia and Hugh-Jones, 1957; Leblanc et al., 1988; Laveneziana et al., 2013a; Guenette et al., 2014; Faisal et al., 2016; Elbehairy et al., 2016).

Current neurophysiological constructs propose that the intensity of dyspnea rises with increasing tidal inspiratory efferent neural activity relative to the maximum possible neural activation (from bulbo-pontine and cortical motor centers in the brain) as indirectly represented by the above physiological ratios (Guenette et al., 2014; Elbehairy et al., 2016; Faisal et al., 2016). Afferent neural inputs from multiple sensory receptors throughout the respiratory system relay precise information concerning the dynamic status of the respiratory system (Caruana-Montaldo et al., 2000; Sant'Ambrogio and Widdicombe, 2001; Widdicombe, 2009). An increase in motor command to ventilatory muscles is perceived as a sensation of respiratory work/effort, or dyspnea (Killian and Jones, 1984), and the increase in ventilation (relative to maximal possible ventilation) required to perform moderate or intense exercise is accompanied by an increasing awareness of increased work or effort of breathing, even in healthy subjects (Killian, 1998). It is further postulated that concomitant increased central corollary discharge from cortical and bulbo-pontine control centers to the somato-sensory cortex, where unpleasant respiratory sensations are consciously perceived, is a final common pathway (Killian et al., 1984; Chen et al., 1992; Banzett et al., 2008).

At exercise termination in both COPD and ILD, central respiratory efferent drive reaches almost maximal values, but the respiratory muscle pump, which is overloaded or functionally weakened, responds inadequately to the increased electrical activation. Thus, despite near maximal drive and effort, very little air enters the lungs with each breath (Figure 7) (Faisal et al., 2016). This disparity is perceived as unpleasant and is the result of central integration of efferent central outputs and multiple afferent peripheral inputs from the respiratory muscles, chest wall and lungs (Carrieri-Kohlman et al., 2001; Widdicombe, 2009). In line with this theory, it has been repeatedly shown that external imposition of mechanical loads to impede respiration in healthy volunteers in the face of increasing chemostimulation reliably provokes respiratory sensations akin to “unsatisfied inspiration” (Figure 7) (O'Donnell et al., 1997, 1998; Faisal et al., 2016). In contrast to obstructive and restrictive lung diseases, dyspnea during activity in PAH appears to be more closely related to the increased inspiratory neural drive reflecting pulmonary gas exchange and cardio-circulatory abnormalities than to deranged mechanics, *per se* (Aguggini et al., 1987).

Although definitive experimental verification is lacking, it is believed that vagal afferent input from the lungs and pulmonary vasculature directly to the somato-sensory cortex, or spinal input from mechanoreceptors in the respiratory muscles and chest wall, can directly induce unpleasant respiratory sensations that shape the clinical expression of dyspnea in all three conditions (Adrian, 1933; Killian et al., 1984; Chen et al., 1992; Chen and Kou, 2000; Banzett et al., 2008; Lee, 2009). Indeed, activation of the pulmonary C fibers and the high-threshold A Delta fibers may alter breathing pattern and contribute to dyspnea (Yu, 2009; Lee and Yu, 2014). Activation of those pulmonary afferents may cause further DH by inducing tachypnea (Yu et al., 1998; Soukhova et al., 1999; Yu, 2000) or by suppression of expiratory muscle activity, thus adding more burdens on the inspiratory muscles (Yu et al., 2001). Notwithstanding, a recent study provides strong evidence that the relationship between increased dyspnea intensity and increased inspiratory neural drive to the diaphragm during exercise is not affected by major disease-specific differences in afferent inputs from the airways, lung parenchyma, chest wall, and respiratory muscles (Figure 7) (Faisal et al., 2016). Additionally, respiratory discomfort beyond a certain threshold evokes emotive responses, such as anxiety, fear, panic or distress. The threshold of the affective distress likely varies between individuals and is thought to be linked to increased activation of limbic and paralimbic centers in the brain and associated over-activation of the sympathetic nervous system (Banzett et al., 2000; von Leupoldt et al., 2008).

## Summary and clinical implications

Based on solid physiological principles established by foundational researchers in the field, such as Wasserman and Whipp (Wasserman et al., 1999), CPET interpretation has traditionally focused on measuring peak $\dot{\text{V}}$O_2_ and has incorporated quantitative assessments of cardiac and ventilatory reserves as well as aerobic capacity. The simple format proposed here extends this approach to include evaluation of perceived intensity of exertional dyspnea and its physiological origins in an individual presenting with respiratory symptoms. This approach is focused on uncovering integrated pathophysiological abnormalities that help explain the origins of dyspnea and exercise intolerance in the individual and is less concerned with diagnostic differentiation. Specifically, analysis of ventilatory efficiency, breathing pattern and operating lung volumes throughout exercise permits a useful non-invasive assessment of pulmonary gas exchange and the prevailing respiratory mechanical constraints which are known to contribute to dyspnea perception. In this context, measurement of operating lung volumes throughout exercise is arguably more sensitive than traditional assessments of breathing reserve ($\dot{\text{V}}$_E_/MVC), at least in patients with earlier or milder COPD and ILD (Dempsey, 2013; Faisal et al., 2016).

The physiological perturbations of early COPD and ILD are generally akin to the effects of accelerated aging: age-related pulmonary gas exchange and mechanical derangements are exaggerated, and inspiratory neural drive and attendant exertional dyspnea become amplified at any given work rate. Indeed, we believe that an exercise challenge test that incorporates the above-outlined measurements allows a comprehensive physiological characterization of pathophysiology in symptomatic patients in the early phases of COPD, ILD, and PAH, whose resting pulmonary function tests are close to the normal range. Using this approach, it is possible to uncover unanticipated abnormalities such as dynamic lung hyperinflation and attendant mechanical constraints, which require further diagnostic evaluation and targeted treatment. For example, the finding of DH in a symptomatic patient with ILD or PAH might lead to a therapeutic trial of a bronchodilator.

Knowledge of the common mechanisms of exertional dyspnea in COPD, ILD, and PAH enables the development of a cogent physiological rationale for personalized management. Thus, interventions that reduce the heightened inspiratory neural drive (exercise training, supplemental O_2_, or opioid medication) can successfully ameliorate dyspnea during physical activity in selected patients. Similarly, interventions that improve respiratory mechanics and dynamic respiratory muscle function (e.g., bronchodilators in COPD, specific inspiratory muscle training in selected patients with measurable weakness) can enhance neuro-mechanical coupling of the respiratory system and improve dyspnea and exercise intolerance. Finally, identification of heterogeneous physiological derangements of pulmonary gas exchange and mechanics during exercise earlier in the course of these common chronic respiratory diseases sets the stage for more precise clinical phenotyping of patients and raises the prospect of developing targeted therapeutic interventions and evaluating their clinical efficacy.

## Author contributions

All authors played a role in the content and writing of all sections of the review. In addition: DO and JN provided the original idea for the review.

## Funding

AE has received financial support through the John Alexander Stewart scholar award from Queen's University. The funders had no role in writing this review. DO, AE, DB, ND, and JN have no conflicts of interest that are directly relevant to the content of this article. Outside the submitted work, DO has received research funding via Queen's University from AstraZeneca, Boehringer Ingelheim and GlaxoSmithKline; and has served on speakers bureaus, consultation panels and advisory boards for AstraZeneca, Boehringer Ingelheim and GlaxoSmithKline.

### Conflict of interest statement

The authors declare that the research was conducted in the absence of any commercial or financial relationships that could be construed as a potential conflict of interest.

## Abbreviations

$\dot{\text{V}}$_A_: Alveolar ventilation
$\dot{\text{V}}$_A_/Q: Ventilation/perfusion
A-aPO_2_: Alveolar-to-arterial oxygen tension gradient
$\dot{\text{V}}$CO_2_: Carbon dioxide production
COPD: Chronic obstructive pulmonary disease
CPET: Cardiopulmonary exercise testing
DH: Dynamic hyperinflation
D_L_CO: Diffusing capacity of the lungs for carbon monoxide
$\dot{\text{V}}$_E_: Minute ventilation
EELV: End-expiratory lung volume
EFL: Expiratory flow limitation
EILV: End-inspiratory lung volume
EMGdi: Diaphragmatic electromyography
ERV: Expiratory reserve volume
*f*: Breathing frequency
FEV_1_: Forced expired volume in one second
FVC: Forced vital capacity
HR: Heart rate
IC: Inspiratory capacity
ILD: Interstitial lung disease
IRV: Inspiratory reserve volume
LV: left ventricle
MVC: Maximal ventilatory capacity
NMD: Neuromechanical dissociation
$\dot{\text{V}}$O_2_: Oxygen consumption
PAP: Pulmonary artery pressure
PaCO_2_: partial pressure of arterial oxygen
PEEP: Positive end-expiratory pressure
Pes: Esophageal pressure
PO_2_: Partial pressure of oxygen
PVR: Pulmonary vascular resistance
RV: Residual volume
TLC: Total lung capacity
VC: Vital capacity
V_D_: Dead space volume
V_T_: Tidal volume
WR: Work Rate PAH: pulmonary arterial hypertension
PetCO_2_: partial pressure of end-tidal carbon dioxide
SV: Stroke volume.
